# An adiponectin-S1P axis protects against lipid induced insulin resistance and cardiomyocyte cell death via reduction of oxidative stress

**DOI:** 10.1186/s12986-019-0342-y

**Published:** 2019-02-21

**Authors:** Amy Botta, Ying Liu, Sivaporn Wannaiampikul, Rungsunn Tungtrongchitr, Keith Dadson, Tae-Sik Park, Gary Sweeney

**Affiliations:** 1 Department of Biology, York University, Toronto, ON M3J 1P3 Canada; 2 Department of Biochemistry, Faculty of Medicine, Srinakharinwirot University, Bangkok, Thailand; 3 Department of Tropical Nutrition and Food Science, Faculty of Tropical Medicine, Mahidol University, Bangkok, Thailand; 4 Department of Life Science, Gachon University, Sungnam, South Korea

**Keywords:** Adiponectin, Sphingosine-1-phosphate, Ceramide, Cardiomyocyte apoptosis, High fat diet, Palmitate, ROS

## Abstract

**Background:**

Adiponectin exerts several beneficial cardiovascular effects, however their specific molecular mechanisms require additional understanding. This study investigated the mechanisms of adiponectin action in the heart during high fat diet (HFD) feeding or in palmitate (PA) treated H9c2 cardiomyoblasts.

**Methods:**

6-week-old male adiponectin knock out (Ad-KO) mice were fed chow or 60% HFD for 6 weeks then received saline or recombinant adiponectin (3μg/g body weight) for an additional 2 weeks. After acute insulin stimulation (4 U/kg), tissue and serum samples were collected for analysis. H9c2 cardiomyocytes were treated ±0.1 mM PA, the adiponectin receptor agonist AdipoRon, or the antioxidant MnTBAP then assays to analyze reactive oxygen species (ROS) production and cell death were conducted. To specifically determine the mechanistic role of S1P, gain and loss of function studies were conducted with adding S1P to cells or the inhibitors THI and SKI-II, respectively.

**Results:**

HFD feeding induced cardiac insulin resistance in Ad-KO mice, which was reversed following replenishment of normal circulating adiponectin levels. In addition, myocardial total triglyceride was elevated by HFD and lipidomic analysis showed increased levels of ceramides and sphingosine-1-phosphate (S1P), with only the latter being corrected by adiponectin administration. Similarly, treatment of H9C2 cardiomyoblasts with PA led to a significant increase of intracellular S1P but not in conditioned media whereas AdipoRon significantly increased S1P production and secretion from cells. AdipoRon or the antioxidant MnTBAP significantly reduced PA-induced cell death. Gain and loss of function studies suggested S1P secretion and autocrine receptor activation mediated the effect of AdipoRon to attenuate PA-induced ROS production and cell death.

**Conclusion:**

Our data establish adiponectin signaling-mediated increase in S1P secretion as a mechanism via which HFD or PA induced cardiomyocyte lipotoxicity, leading to insulin resistance and cell death, is attenuated.

## Background

Obesity is a major risk factor for the development of cardiovascular diseases, including heart failure [1, 2]. Previous studies have identified many contributors to the initiation and progression of cardiac remodeling in obesity including lipotoxicity, hypoadiponectinemia, and insulin resistance [3–5]. Lipotoxicity is a consequence of a high-fat diet (HFD), resulting in elevated circulating free fatty acids and can lead to insulin resistance and metabolic dysfunction [6]. Altered cardiac energy metabolism is well known as one of the first observable cardiac remodeling events during development of heart failure [7]. Metabolic changes include less mitochondrial oxidative metabolism, elevated glycolysis, as well as uncoupling between glycolysis and glucose oxidation. Collectively these result in metabolic inefficiency leading to cardiac contractile dysfunction. Insulin resistance is well characterized as a major contributor to cardiac dysfunction via metabolic and other cellular consequences [8]. Accordingly, metabolic modulation, such as via insulin sensitization, remains a priority target for new therapeutics [9]. In addition, cardiomyocyte apoptosis is another important consequence of lipotoxicity which contributes to the development of heart failure [10, 11].

Circulating adiponectin (Ad) levels correlate negatively with visceral obesity, diabetes and cardiovascular diseases [5]. Adiponectin has been shown to have beneficial cardioprotective effects, including direct metabolic, insulin-sensitizing, and anti-apoptotic effects [5]. Ad acts via binding to Ad receptor 1 (AdipoR1) and 2 (AdipoR2) [12]. Early studies identified two Ad receptor adapter proteins adaptor protein, phosphotyrosine interacting with PH domain and leucine zipper 1 (APPL1) and 2 (APPL2) which regulate the downstream activation of effectors such as AMP-activated protein kinase (AMPK) [13], leading to increased glucose uptake and lipid uptake and oxidation. Indeed, transgenic mice overexpressing APPL1 were protected from HFD-induced cardiomyopathy [14]. Insightful studies have identified AdipoR-mediated activation of ceramidase activity [15] as leading to enhanced ceramide conversion to S1P. This direct link of lipid signaling and metabolism has subsequently been shown to have important metabolic consequences in glucose and lipid metabolism [16, 17].

Since Ad can elicit various cardioprotective cellular effects, there has been great interest within the pharmaceutical industry to identify small molecules which act as AdipoR agonists [18, 19]. One such compound, AdipoRon, was identified and shown to mimic Ad signaling in various cell types and animal models [20, 21]. However, the exact mechanisms via which Ad or AdipoRon protect the heart during diet-induced obesity and cardiomyocytes from lipotoxic conditions require further research to provide additional insight. Here we used Ad knockout (Ad-KO) mice to examine cardiac insulin sensitivity and lipid metabolism after HFD feeding. We also examined the role of Ad in correcting HFD-induced abnormalities in lipid metabolism and insulin action by replenishing normal circulating Ad levels in one group of Ad-KO mice. We then studied potential cellular mechanisms using a model of lipid-induced oxidative stress and cell death in H9c2 cardiomyoblasts, with or without AdipoRon treatment. Our data provide new insights into the mechanisms of HFD induced cell death and further highlight the role of Ad and S1P in these mechanisms.

## Methods

### Materials

Insulin (Humulin R) was purchased from Eli Lilly (Toronto, Canada). Polyclonal phosphospecific antibodies to Akt (T308&S473), total Akt, pAMPK Thr172, AMPK, GAPDH and horseradish peroxidase (HRP)-conjugated anti-rabbit-IgG were from Cell Signaling Technology (Beverly, MA), while polyclonal phospho-specific antibodies to AdipoR1 and AdipoR2 were a kind gift from Dr’s Tony Clementz and Jan Oscarsson (Astra Zeneca, Sweden), and APPL1, APPL2 antibodies from Antibody Immunoassay Services (Hong Kong). Polyvinylidene difluoride (PVDF) membrane was from Bio-Rad (Burlington, ON) and chemiluminescence reagent plus was from PerkinElmer (Boston, MA). 2-acetyl-5-tetrahydroxybutyl imidazole (THI), an inhibitor of sphingosine-1-phosphate lyase, and 4-[4-(4-chloro-phenyl)-thiazol-2-ylamino]-phenol (SKI-II), an inhibitor of sphingosine kinase, were purchased from Cayman Chemical (Ann Arbor, MI). S1P and MnTBAP were purchased from Sigma Aldrich (St. Louis, MI). All other reagents and chemicals used were of the highest purity available.

### Experimental animals

Male Ad-KO mice [22] bred and genotyped in-house were randomly allocated to experimental groups as indicated. At 6 weeks of age, animals were fed either regular chow diet or 60% high-fat diet (HFD) for a period of 6 weeks. At the end of the 6 weeks, chow and HFD-fed Ad-KO animals received either saline or Adiponectin (3 μg/g body weight) twice daily for an additional 2 weeks via intraperitoneal injection. This approach has been optimized as the amount required to restore circulating adiponectin levels to within the normal range [23–25]. Ad was prepared in saline as described previously [26]. At the end of the treatment, animals were starved for 5–6 h and were then subjected to acute insulin stimulation by a bolus insulin (4 U/kg body weight) injection via tail vein; tissues were harvested 15 min later. Tissue samples were collected and snap frozen in liquid nitrogen (N_2_) and kept together with serum samples from the same animals at − 80 °C until analysis.

### Heart homogenization and analysis

Whole hearts were snap frozen in liquid nitrogen and prepared for analysis as described previously [27]. Tissue-specific TG and ATP content were analyzed using the colorimetric Triglyceride Quantification and ATP Assay Kit, whereas lactate content was evaluated using the Lactate Colorimetric Assay Kit II purchased from Biovision (California, USA).

#### Preparation of heart homogenates, cell lysates, and Western blotting

All tissue and cell samples were prepared as described previously [28] and primary antibodies [phospho-Akt (S473&T308), total Akt, AdipoR1, AdipoR2, APPL1, APPL2 and GAPDH] were incubated for 1 h at a dilution of 1:1000. Membranes were then washed four times with 1x wash buffer for 15 min each at room temperature and incubated with the appropriate HRP-coupled secondary antibody (1,10,000). Membranes were washed five times with 1x wash buffer for 10 min each and proteins visualized using enhanced chemiluminescence. Quantitation of each specific protein band was then determined via densitometric scanning with correction for the respective loading control.

### Lipidomic analysis of myocardial lipids

Cardiac diacylglycerols (DAGs) were measured by LC-MS-MS with atmospheric pressure chemical ionization (APCI) source and measured species were 16:0, 18:1, 18:0–20:4, 16:0–18:1, and 18:0–18:2 DAGs using HPLC 1200 series (Aglient Technologies, Santa Clara, CA, USA) with a Gemini C6-phenyl column (50 × 2.0 mm i.d., 3 μm, Phenomenex, Torrance, CA, USA) and Triple Quadrupole/Ion Trap mass spectrometer equipped with Heated Nebulizer interfaces (4000 QTrap, ABSciex, Foster city, CA) as described previously [29]. Ceramides with various acyl chains (C14:0, C16:0, C18:0, C18:1, C20:0, C24:0, C24:1) were separated by HPLC with a C18 column (XTerra C18, 3.5 m, 2.150 mm) and ionized in positive electrospray ionization mode as described previously [30]. Sphingolipid metabolites were monitored for multiple reaction monitoring (MRM) quantification by a bench-top tandem mass spectrometer with an electrospray ionization source.

### S1P ELISA kits

For both heart and serum samples, the level of S1P was measured using an ELISA from Echelon Biosciences (Salt Lake City, UT), following manufacturers instructions. As this kit is not suitable for detection for S1P from rat based samples, for cell culture experiments with H9c2 cells a rat-specific S1P ELISA kit was purchased and conducted following manufactures instructions from MyBioSource (San Diego, CA).

### Fatty acid preparation

A Stock solution of palmitate (PA) was prepared by dissolution in 70% ethanol to create a 100 mM concentration stock. This solution was then diluted with 8% bovine serum albumin (BSA) in PBS to make 5 mM working solutions. A control BSA solution was prepared by using an equal amount of 70% ethanol to that of the PA solution. Conjugation was completed at 37 °C with shaking for 4 h. Finally, the 5 mM stock solutions were sterile filtered into aliquots and stored at -80 °C.

### H9c2 cardiomyocyte experiments and analysis

H9c2 cells were plated in a 96 well plate (50,000 cells/ml) and left overnight. Cells were then incubated with 0.5% BSA for 4 h. Following which 0.1 or 0.25 mM concentrations of either BSA or PA were added. Additional a subset of cells was then treated with either 35 μM AdipoRon, 100 μM MnTBAP, 5 μM SKI-II, 5 μM THI, or 2.5 μM S1P as indicated. For the DCF-DA assay cells were first washed with PBS++ and then incubated with 20 μM DCF-DA for 30 min. Cells were then washed twice in PBS++. Following which fluorescence was measured at 488 nm excitation and 525 nm emission.

The LDH release assay was conducted using a kit following manufacturers instructions (G-Biosciences, St. Louis, MO). The Trypan Blue plate based assay was conducted according to a previously published protocol [31]. Briefly trypan blue was added to the wells at a final concentration of 0.05% and incubated for 15 min. Following which the wells were washed gently using 300ul PBS++ three times. Then 100ul of a 1% SDS solution was added to each well and incubated on the shaker for 5 min to ensure lysis. The plate was then read at 590 nm. The Caspase 3/7 assay was conducted using the CellEvent Caspase 3/7 Green Detection reagent manufacturers instructions (Thermofisher, Waltham, MA).

### Statistical analysis

All data were calculated as means ± SEM and further analyzed using one-way ANOVA or Welch’s two-sample *t*-tests as appropriate. Differences were considered statistically significant at *P* < 0.05 and for lipidomics were further stratified to *P* < 0.01 and *P* < 0.001 respectively.

## Results

### Replenishment of adiponectin in ad-KO mice corrects high fat diet-induced cardiac insulin resistance

**Fig. 1:**
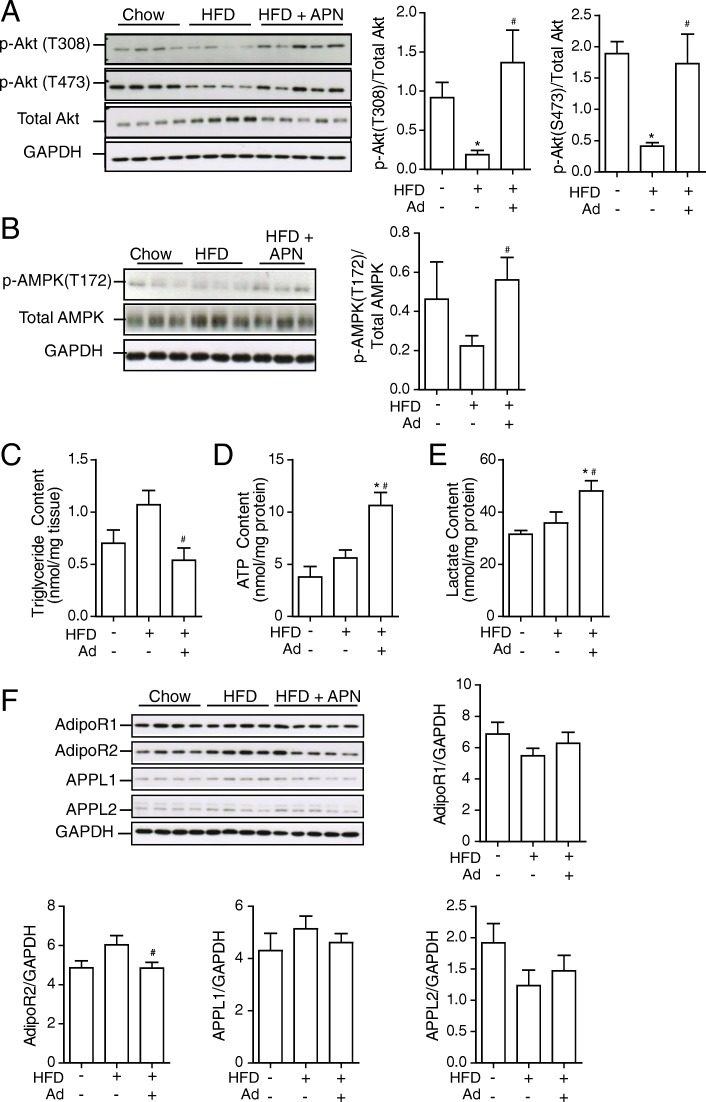
Adiponectin treatment reverses diet-induced cardiomyocyte insulin resistance. Adiponectin knockout (Ad-KO) mice were fed either commercial chow (Chow) or 60% high fat diet (HFD) diet at the age of 6 weeks for a period of 6 weeks. After 6 weeks of HF diet, Ad-KO mice were treated with either saline or Ad at a dosage of 3 μg/g body weight twice a day for additional 2 weeks via intraperitoneal injection. Insulin signaling was assessed 15 min after a bolus injection of insulin (4 U/kg body weight) via the tail vein upon 4–5 h of fasting. **a** Representative western blot images and quantitative analysis of **a**) insulin-stimulated phosphorylation of Akt, at both Thr308 and Ser473 compared to total Akt and **b**) p-AMPK (thr172) over total AMPK. **c** Triglyceride, **d** ATP and **e**) Lactate content were analyzed in whole heart tissue collected from animals. **f** Expression levels of AdipoR1, AdipoR2, APPL1 and APPL2 from heart tissues collected from Ad-KO mice with or without additional adiponectin treatment upon chow or HFD. GAPDH was used as loading control. Data represent mean ± SEM; *P < 0.05 vs Chow, ^#^*P* < 0.05 vs 60% HFD, *n* = 4–5

Insulin-stimulated signaling (Akt T308 and S473) was examined in the heart of Ad-KO mice fed chow or HFD for 6 weeks and in the latter group also after administration of Ad for an additional 2 weeks (Fig. 1a). Ad-KO mice on HFD had significantly reduced insulin sensitivity compared to chow-fed Ad-KO mice, which was reversed following treatment with Ad (Fig. 1a). In addition, restoring Ad levels in Ad-KO mice led to an increase in phospho-AMPK levels similar to that observed in chow-fed Ad-KO controls (Fig. 1b). Furthermore, restoring normal circulating Ad levels lowered myocardial triglyceride content (Fig. 1c), and increased ATP and lactate content (Fig. 1d&e). These Ad-induced changes were not correlated with any significant changes in AdipoR1 the adaptor proteins APPL1 and APPL2, however, there was a reduction in AdipoR2 protein levels after administration of Ad (Fig. 1f).

### Lipidomic analysis of metabolite profiles in ad-KO mice fed chow or HFD with and without adiponectin replenishment

**Fig. 2:**
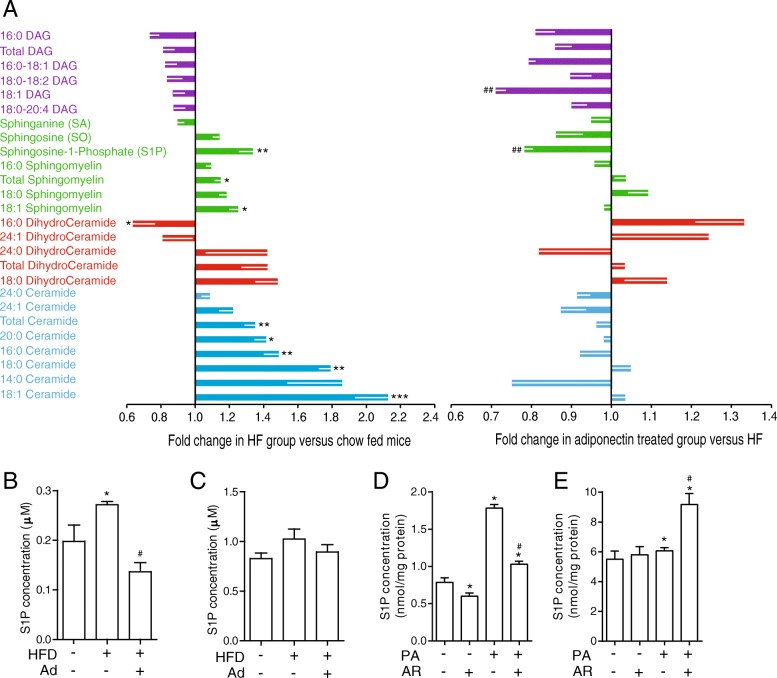
Lipidomic analysis of HFD-induced myocardial changes in Ad-KO mice and the effect of adiponectin replenishment. **a** Lipidomic analyses were performed on heart tissues collected from Ad-KO mice treated as described in Fig. 3 and data are shown for the myocardial content of different species of diacylglycerols, sphingomyelins, and ceramides. Left panel: influence of HFD versus chow is shown as fold change observed in HF group versus chow-fed mice. Right panel: graph shows fold change in adiponectin-treated versus saline-treated within HFD group. Changes indicate a corrective effect of adiponectin. Data represent mean ± SEM; * significant difference between HF diet group and chow diet group; # significant difference between Ad and saline treatment within HF diet group; *,#, *P* < 0.05; **,##, *P* < 0.01; ***,###, *P* < 0.001, *n* = 5–6. The concentration of S1P in **b**) Heart (intracellular S1P) and **c**) Serum (extracellular S1P) in Ad-KO animals fed either a chow, 60% HFD or a 60% HFD plus Ad at a dosage of 3 μg/g for two weeks adiponectin using an ELISA kit. Data represent mean ± SEM, *P < 0.05 vs WT, ^#^*P* < 0.05 vs HF, *n* = 3–5. **d** The concentration of S1P in H9c2 cells treated (intracellular S1P) and **e**) the media of these cells (extracellular S1P) with either 0.1 mM PA, with and without 35 μM AdipoRon (AR). *P < 0.05 vs BSA, ^#^*P* < 0.05 vs PA, *n* = 4

Lipidomic analysis was performed to profile specific lipid species in hearts isolated from Ad-KO mice after 6 weeks feeding with chow or HFD ± Ad replenishment for 2 weeks. In Fig. 2a, the left side vertical axis represents data comparing changes in Ad-KO mice on HFD versus chow diet (decrease to left and increase to right). HFD significantly increased the production of total ceramides, in particular several distinct ceramide species (16:0, 18:1, 18:0 and 20:0) were most significantly increased (Fig. 2a). Total and 18:1 sphingomyelin was also increased by HFD (Fig. 2a). Somewhat surprisingly, these were not significantly altered after Ad replenishment as shown on Fig. 2a right side vertical axis. Notably, a decreased 16:0 dihydroceramide level was observed in response to HFD and this was largely reversed by Ad (Fig. 2a). Importantly, sphingosine-1-phosphate (S1P) content in the heart was increased by HFD and significantly reversed by Ad (Fig. 2a). To gain further insight into these changes, we next examined S1P in serum and myocardium of these mice using an ELISA-based approach and found similar observations in heart tissue, where there was an increased in intracellular S1P (Fig. 2b) whilst there were no significant alterations in serum, which represents extracellular levels of S1P (Fig. 2c). To translate the observations in mice ± HFD to an in vitro model, we next tested S1P levels by ELISA in H9c2 cells treated with palmitate (PA). An increase in intracellular (H9c2 cells), but not extracellular (cellular media), S1P was observed after PA treatment (Fig. 2d&e). Addition of AdipoRon elicited release of S1P resulting in an increase in the concentration of S1P in the media (Fig. 2d&e).

### Pharmacological manipulation of S1P levels or addition of AdipoRon regulates ROS production in palmitate-treated H9c2 cells

**Fig. 3:**
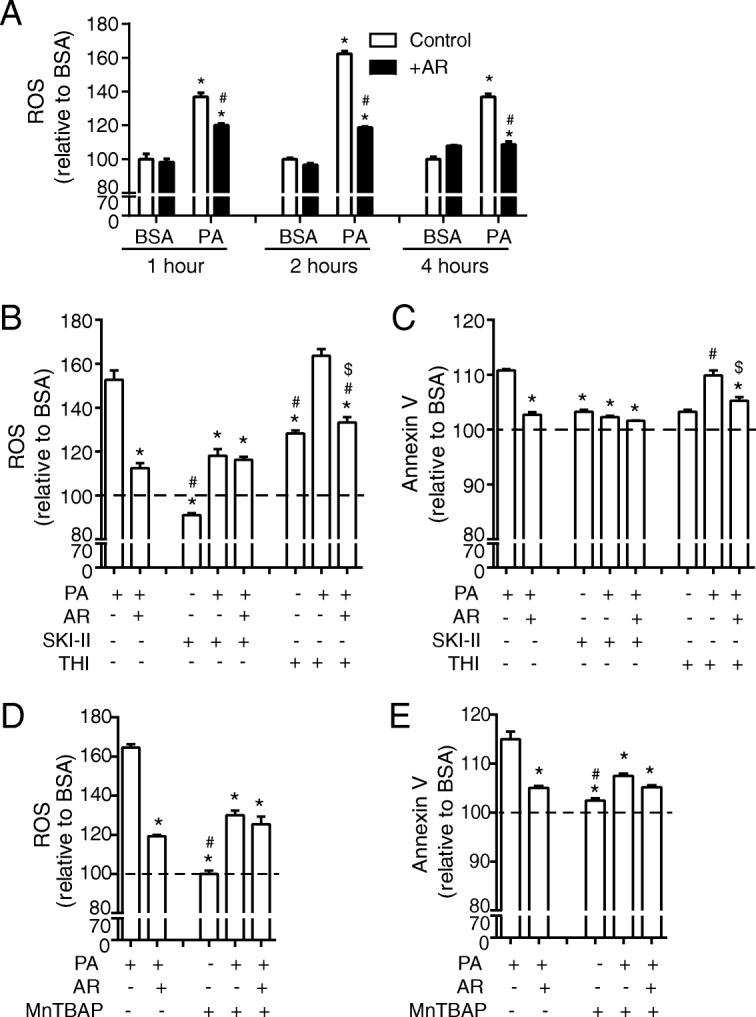
Pharmacologic regulation of S1P levels significantly alters ROS production in H9c2 cells treated ± PA. **a** ROS production in H9c2 cells measured using a DCF-DA assay. Following a preincubation with 0.5% FBS, cells were incubated with 0.1 mM FA for 1, 2, or 4 h or were also treated with the addition of 35 μM AdipoRon (AR) as indicated. Data represent mean ± SEM; *P < 0.05 vs BSA, ^#^*P* < 0.05 vs PA, *n* = 6. In a subset of cells in addition to 0.1 mM PA or a BSA vehicle control with or without the addition of 35 μM AR, 5 μM SKI-II and 5 μM THI were added for 4 h following which **b**) ROS was measured using DCF-DA assay and **c**) cell death was measured using an Annexin V assay (shown as % toxicity). *P < 0.05 vs PA, ^#^*P* < 0.05 vs PA + AR, ^$^*P* < 0.05 vs PA + THI, *n* = 3. In a subset of cells 100 μM MnTBAP was added for 4 h following which **d**) ROS was measured using DCF-DA assay and **e**) cell death was measured using an Annexin V assay (shown as % toxicity). Data represent mean ± SEM; *P < 0.05 vs PA, ^#^*P* < 0.05 vs PA + AR, *n* = 3

Addition of PA to H9c2 cells for 1, 2 and 4 h significantly increased levels of reactive oxygen species (ROS), and this was significantly attenuated by AdipoRon (Fig. 3a). To determine the role of S1P in the modulation of the PA-induced ROS response we used sphingosine kinase inhibitor SKI-II to prevent S1P production in response to PA, and this significantly reduced ROS production (Fig. 3b). S1P-lyase catalyzes the irreversible decomposition of S1P to trans-2-hexadecenal and phosphoethanolamine and its inhibitor, THI, caused a small but not significant additive effect with PA on ROS production (Fig. 3b). In cells treated with BSA, incubation with SKI-II did not significantly increase ROS production compared to BSA control, however the addition of THI did significantly increase ROS production in comparison to BSA (Fig. 3b). Annexin V staining showed that there was an increase in apoptosis after treatment with PA that was attenuated by the addition of AdipoRon or SKI-II (Fig. 3c). The addition of THI significantly increased cell death (Fig. 3C). Finally, we used the antioxidant MnTBAP and confirmed that it effectively prevented PA-induced ROS to a level similar to that observed with AdipoRon, and not in an additive manner (Fig. 3d). Annexin V staining after 4 h showed that there was an increase in apoptosis after treatment with PA that was attenuated by the addition of AdipoRon or MnTBAP (Fig. 3e).

### AdipoRon and pharmacological manipulation of ROS decreases palmitate-induced cell death

**Fig. 4:**
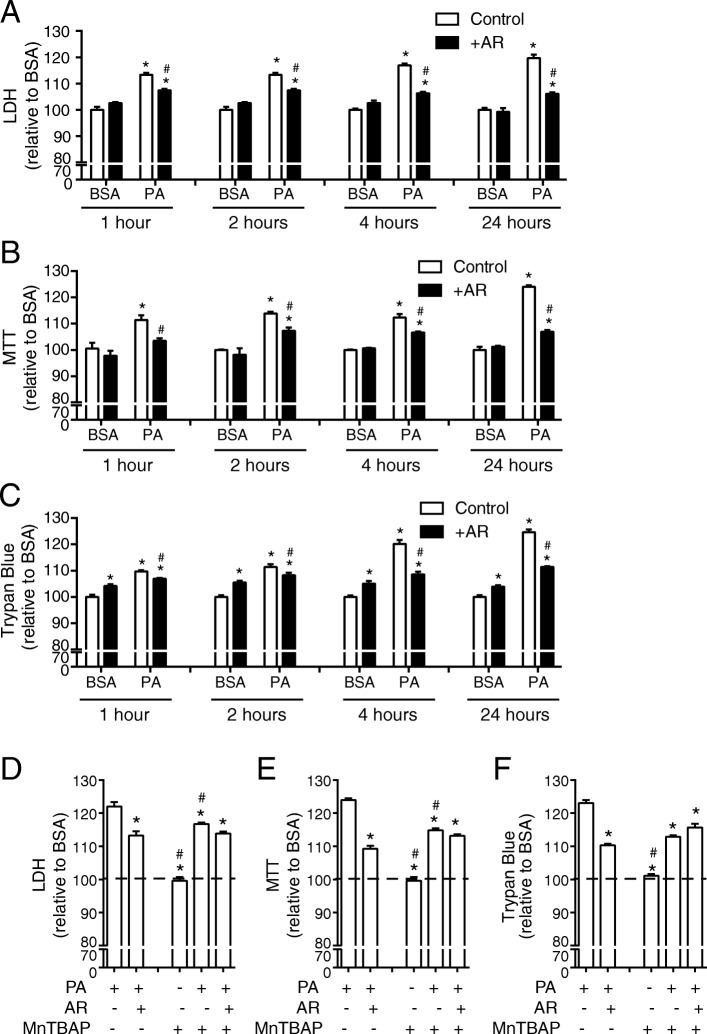
Reducing PA-induced ROS production with MnTBAP or AdipoRon attenuates cell death in H9c2 cells. Cell death in H9c2 cells measured using a **a**) LDH, **b**) MTT or **c**) Trypan blue assay. Following a preincubation with 0.5% FBS, cells were incubated with 0.1 mM FA for 1, 2, or 4 h or were also treated with the addition of 35 μM AdipoRon (AR) as indicated. For all graphs the y-axis is displayed as % toxicity relative to BSA. Data represent mean ± SEM; *P < 0.05 vs BSA control group, ^#^*P* < 0.05 vs PA control group, *n* = 6. In another subset of cells 100 μM MnTBAP was added for 24 h following which **d**) LDH, **e**) MTT or **f**) Trypan blue was measured. For all graphs the y-axis is displayed as % toxicity relative to BSA. Data represent mean ± SEM; *P < 0.05 vs PA, ^#^*P* < 0.05 vs PA + AR, *n* = 3

To further confirm that cell death is a major consequence of elevated ROS in PA-treated H9c2 cells, we measured this by LDH (Fig. 4a), MTT (Fig. 4b) and trypan blue assays (Fig. 4c). AdipoRon alone caused no significant change in cell death assessed using LDH and MTT assays (Fig. 4a, b) and a small but significant increase when trypan blue assay was used (Fig. 4c). Importantly, AdipoRon significantly attenuated PA-induced cell death (Fig. 4a-c). We next used the antioxidant MnTBAP and confirmed that it effectively prevented PA-induced cell death to a level similar to that observed with AdipoRon (Fig. 4d-f). Across all measures of PA-induced cell death, there was a similar extent of attenuation by AdipoRon and MnTBAP, and effects of these two compounds were not additive (Fig. 4d-f).

### AdipoRon and pharmacological manipulation of S1P protect H9c2 cells from palmitate-induced cell death

**Fig. 5:**
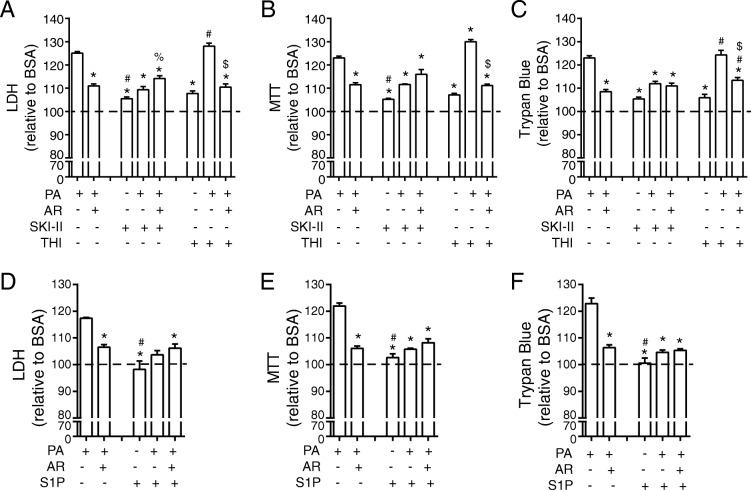
Manipulation of S1P action significantly alters cell death in H9c2 cells. Cell death in H9c2 cells measured using a **a**) LDH, **b**) MTT or **c**) Trypan blue assay. Following a preincubation with 0.5% FBS, cells were incubated with 0.1 mM FA for 24 h and were also treated with the addition of 35 μM AdipoRon (AR), 5 μM SKI-II and 5 μM THI as indicated. For all graphs the y-axis is displayed as % toxicity relative to BSA. Data represent mean ± SEM; *P < 0.05 vs PA, ^#^*P* < 0.05 vs PA + AR, ^%^*P* < 0.05 vs PA + SKI-II, ^$^*P* < 0.05 vs PA + THI, n = 3. In another subset of cells 2.5 μM S1P added for 24 h following which **d**) LDH, **e**) MTT or **f**) Trypan blue was measured. For all graphs the y-axis is displayed as % toxicity relative to BSA. Data represent mean ± SEM; *P* < 0.05 vs PA, ^#^*P* < 0.05 vs PA + AR, *n* = 3

Similar to incubation with the antioxidant MnTBAP, various measures of cell death and viability including LDH assay (Fig. 5a), MTT assay (Fig. 5b), and trypan blue assay (Fig. 5c) all indicated that SKI-II attenuated PA-induced cell death. Incubation of BSA treated cells with SKI-II did not significantly increase cell death compared to cells treated with BSA and AdipoRon. Similarly, in cells treated with PA, the effect of SKI-II was similar in magnitude to the effect of AdipoRon and not additive (Fig. 5a-c). In these assays, THI did not significantly alter the effect of PA or the ability of AdipoRon to attenuate PA-induced cell death (Fig. 5a-c). Increasing S1P receptor activation directly through exogenous addition of S1P, led to a significant reduction in cell death as measured by LDH (Fig. 5d), MTT (Fig. 5e) and trypan blue (Fig. 5f). Co-incubation with S1P and AdipoRon was not additive (Fig. 5d-f).

## Discussion

A large body of published literature has demonstrated the correlations between obesity and myocardial infarction or development of heart failure. Furthermore, the underlying mechanisms have been extensively studied [32, 33]. To do so, high-fat feeding in animal models or use of high levels of fatty acids in cultured cells have often been employed. Consequently, numerous cardiac remodeling processes are now known to occur which increase the risk of myocardial infarction or promote the progressive development of heart failure [2]. Altered metabolism is often one of the earliest and most important changes which occurs in obesity [7, 34]. This typically involves reduced glucose metabolism and an unfavourable switch toward over-reliance of fatty acids as a substrate for energy production [7, 34]. This may occur at least in part due to development of insulin resistance, which is now well established as a major potential contributor to cardiac disease [8]. Another myocardial remodeling event which occurs in lipotoxic models, with or without insulin resistance, is cardiomyocyte cell death [35]. For many years our lab has studied the cardioprotective actions of Ad and the mechanisms via which Ad acts [5, 36]. In this study, we used both animal and in vitro models of lipotoxicity to examine mechanisms leading to insulin resistance or cell death and how these are regulated by Ad. The development of cardiac dysfunction in HFD fed mice requires approximately 16 weeks [37, 38], and since the focus of this research was on evaluating the early mechanisms leading to insulin resistance and metabolic consequences, 6 weeks was chosen [2, 14]. Increased accumulation of myocardial lipids due to HFD, is known to induce cardiac insulin resistance and metabolic dysfunction [32, 33]. Here we used this approach in Ad-KO mice and observed elevated total lipid accumulation in Ad-KO after HFD feeding which was correlated with faster and exaggerated myocardial insulin resistance. Specifically, our lipidomic analysis of mouse hearts led us to focus on the significance of S1P, as discussed below.

Of the specific lipids elevated in high-fat fed Ad-KO mice, there was a significant increase in total and numerous ceramide species, including 14:0, 16:0, 18:0, 18:1, 20:0. However, there was no significant increase in the ceramide precursor dihydroceramide. Previous research has indicated that both ceramide and dihydroceramide may play a role in insulin sensitivity [39, 40], and that increased levels of total dihydroceramide may be potential biomarker for diabetes susceptibility [41]. These data were expected based on previous literature [42, 43]. Although our original hypothesis was that adiponectin would correct these elevated ceramide levels, this did not occur. Additionally, replenishment of adiponectin slightly increased total dihydroceramide, however this increase was not significant. Here we found a significant increase in S1P, which is produced from ceramide via ceramidase and sphingosine kinase activity [44], and this was significantly reduced when normal circulating Ad levels were restored. This was a striking observation in light of literature indicating that Ad interaction with its cellular receptors stimulated ceramidase activity and increased S1P production [15, 45]. Several studies have now shown that S1P mediates the actions of Ad, including SERCA2 activation via CaMKII-PLB signaling [46]. Although the above reduction in myocardial S1P after Ad replenishment may at first appear paradoxical, it is important to note that S1P acts via secretion and autocrine/paracrine activation of its cell surface receptors [47]. This was highlighted from our in vitro studies where we detected an increased S1P production and secretion in response to AdipoRon, whereas PA increased intracellular but not extracellular S1P. A limitation of our study is that ceramidase activity was not directly measured, but the well established literature showing adiponectin receptors contain intrinsic ceramidase activity [45, 48], and the metabolite changes we observed reassure that adiponectin or AdipoRon do stimulate this enzymatic activity. We thus decided to next investigate the significance of S1P secretion and its promotion by Ad action in PA-induced lipotoxicity.

To directly study the significance of S1P in this in vitro model we added S1P to cell culture media to directly stimulate S1P receptors and used the S1P-lyase inhibitor THI, both to confer a gain of function. We also used the sphingosine kinase inhibitor SKI-II to prevent S1P production as a loss of function approach. Our data were largely in agreement with previous research showing that increasing receptor-mediated S1P action through exogenous addition of S1P led to a decreased PA-induced apoptosis in primary cardiomyocytes [15]. Indeed, many examples demonstrating beneficial effects of S1P signaling in preventing cell death in various cells or tissues now exist [49, 50] although, paradoxically, adverse effects have also been reported [51]. Moreover, one previous study showed that PA increased S1P production in hepatocytes and, unlike our data in H9c2 cells, it was released into the extracellular environment leading to S1P3 receptor activation [52]. Another study suggested that S1P activated the S1P2 receptor to impair hepatocyte insulin signaling [53]. Treatment of mice with a sphingosine kinase inhibitor significantly decreased S1P levels leading to decreased plasma insulin levels [54]. However, in combination with inhibition of Akt signaling, increased levels of S1P resulted in decreased insulin production as a result of signaling through S1P2 receptor [55]. Importantly, overexpression of sphingosine kinase 1 leads to increased production of S1P and a reduction in insulin resistance in skeletal muscle after HFD compared to controls [56]. After pharmacologically increasing intracellular levels of S1P, as does PA, we observed a slight increase in ROS and apoptosis. This is consistent with previous findings which showed that increased levels of S1P, led to increased production of the S1P metabolite trans-2-hexadecenal which is cytotoxic [57]. This indicates that increasing production and secretion of S1P or exogenous stimulation of S1P receptors can elicit cardioprotective effects, whereas intracellular accumulation of S1P can be lipotoxic. We showed that the addition of AdipoRon alone increases the production and export of S1P and that AdipoRon stimulated the release of intracellular S1P accumulated after PA treatment, thereby mediating protective effects against ROS production and cell death.

It has been shown that in ischemia-reperfusion ROS increases levels of ceramide and also leads to the degradation of sphingosine kinase 1 and 2, enzymes responsible for the synthesis of S1P [58]. Additionally, previous research has shown that addition of antioxidants such as MnTBAP or NAC [59, 60] after HFD can reduce insulin resistance [61, 62]. We used gain and loss of function studies to show that extracellular S1P is able to attenuate PA-induced ROS and apoptosis, whereas in the absence of AdipoRon increasing cellular S1P increases ROS and cell death. Our findings are in agreement with previously published studies which show that addition of exogenous S1P protects both isolated hearts, cardiomyocytes, and endothelial cells from ROS induced apoptosis [63–65]. However, after pharmacologically increasing levels of S1P, we observed an increase in apoptosis and cell ROS. This is likely due to an inability of S1P to be transported out of the cell. This is consistent with previous findings which showed that increased levels of S1P led to increased production of the S1P metabolite trans-2-hexadecenal which has previously been shown to be cytotoxic, and led to increased levels of ROS. This was attenuated with the addition of AdipoRon which caused a release of S1P from the cell and a reduction in ROS [57]. AdipoRon interacts with the AdipoR1) and 2 (AdipoR2) receptors increasing their intrinsic ceramidase activity, activating the S1P conversion pathway and thereby preventing the buildup of more toxic lipid species [15, 45, 66]. While it is expected that adiponectin will alter insulin sensitivity in HFD fed animals and in cardiomyocytes [24, 25, 67–69] in the in vitro model insulin was not added, and thus altered insulin sensitivity is not a contributing factor to the observed anti-apoptotic effects. Previous literature has shown that a decrease in antioxidant capacity in the heart, leads to increased ROS generation and can lead to the development of cardiomyopathy [70]. We have shown previously that Ad enhanced antioxidant potential in skeletal muscle to alleviate lipid-induced ROS production [68].

## Conclusions

Using both an in vivo mouse model of HFD in wt or Ad-KO mice ± Ad replenishment and cultured H9c2 cells treated with PA ± AdipoRon, our data indicate that S1P is an important mechanistic route via which adiponectin may provide cardioprotection via improving cardiomyocyte insulin sensitivity as well as reducing ROS production and cell death. This data provides further support for a cardioprotective adiponectin receptor-S1P axis and efforts to translate these observations for therapeutically benefit are likely to be of great potential [71–73].

## Abbreviations

Ad: Adiponectin
AdipoR: Adiponectin receptor
AdipoR1: Adiponectin receptor 1
AdipoR2: Adiponectin receptor 2
Ad-KO: Adiponectin Knockout
AMPK: AMP-activated protein kinase
APCI: Atmospheric pressure chemical ionization
APPL1: Adaptor protein, phosphotyrosine interacting with PH domain and leucine zipper 1
APPL2: Adaptor protein, phosphotyrosine interacting with PH domain and leucine zipper 2
AR: AdipoRon
BSA: Bovine serum albumin
DAGs: Diacylglycerols
HFD: High-fat diet
MRM: Multiple reaction monitoring
PA: Palmitate
ROS: Reactive Oxygen Species
S1P: Sphingosine-1-phosphate
SKI-II: 4-[4-(4-chloro-phenyl)-thiazol-2-ylamino]-phenol
THI: 2-acetyl-5-tetrahydroxybutyl imidazole
